# Auditory evoked potential P300 in adults: reference values

**DOI:** 10.1590/S1679-45082016AO3586

**Published:** 2016

**Authors:** Dayane Domeneghini Didoné, Michele Vargas Garcia, Sheila Jacques Oppitz, Thalisson Francisco Finamôr da Silva, Sinéia Neujahr dos Santos, Rúbia Soares Bruno, Valdete Alves Valentins dos Santos Filha, Pedro Luis Cóser

**Affiliations:** 1 Universidade Federal de Santa Maria, Santa Maria, RS, Brazil.; 2 Clínica Cóser, Santa Maria, RS, Brazil.

**Keywords:** Evoked potentials, auditory, Event-related potentials, P300, Adult

## Abstract

**Objective:**

To establish reference intervals for cognitive potential P300 latency using tone burst stimuli.

**Methods:**

This study involved 28 participants aged between 18 and 59 years. P300 recordings were performed using a two-channel device (*Masbe*, Contronic). Electrode placement was as follows: Fpz (ground electrode), Cz (active electrode), M1 and M2 (reference electrodes). Intensity corresponded to 80 dB HL and frequent and rare stimulus frequencies to 1,000Hz and 2,000Hz, respectively. Stimuli were delivered binaurally.

**Results:**

Mean age of participants was 35 years. Average P300 latency was 305ms.

**Conclusion:**

Maximum acceptable P300 latency values of 362.5ms (305 + 2SD 28.75) were determined for adults aged 18 to 59 years using the protocol described.

## RESUMO

**Objetivo:**

Estabelecer valores de referência para a latência do potencial cognitivo P300 com estímulos *tone burst*.

**Métodos:**

Participaram do estudo 28 indivíduos entre 18 e 59 anos. O registro do P300 foi realizado no equipamento Masbe da marca Contronic. Os eletrodos foram fixados nas posições Fpz (eletrodo terra), Cz (eletrodo ativo), M1 e M2 (eletrodos referência). A intensidade foi de 80 dB NA. A frequência do estímulo frequente foi de 1.000Hz e a do estímulo raro de 2.000Hz. Os estímulos foram apresentados na forma binaural.

**Resultados:**

A média de idade dos indivíduos foi de 35 anos. A média de latência para P300 de 305ms.

**Conclusão:**

Usando o protocolo descrito, o valor máximo de latência aceitáveis para P300 foram de 362,5ms (305 + 2DP 28,75) na faixa etária do adulto de 18 a 59 anos.

## INTRODUCTION

Long latency auditory evoked potentials (LLAEPs) can be used in clinical practice as an objective measure of cognitive processes to assess auditory abilities, such as discrimination, memory, attention and detection of stimuli. Long latency AEPs are thought to be one of the most promising electrophysiological tests for evaluation of central auditory nervous system (CANS) dysfunction and/or changes.

Long latency AEPs enable the assessment of auditory information processing over time.^(1)^ These potentials are generated by several systems, primarily the thalamocortical and corticocortical auditory pathways, the primary auditory cortex and associative cortical areas,^(2)^ and reflect thalamic and cortical activity in particular. Long latency AEPs result from the superposition of all electrical currents within the brain; therefore, accurate identification of neural generators is difficult. Neural generators are simultaneously activated and each individual CANS structure is responsible for the processing of one specific aspect of a given stimulus or information.^(3)^

Long latency AEPs are represented by a series of positive and negative waves.^(4)^ While some LLAEPs are sensitive to physical characteristics of the eliciting stimulus (exogenous potentials P1, N1, P2 and N2), others reflect cognitive processes, such as attention and stimulus categorization (endogenous potential P300).^(5)^

The P300 component, or cognitive potential, is a positive potential elicited by the recognition of a rare stimulus (oddball paradigm) within a series of frequent stimuli and corresponds to the largest positive wave after the N1-P2 complex.^(6,7)^ P300 depends upon some abilities, such as attention, discrimination and memory, and reflects cortical activity.^(8)^

P300 latency is the most commonly used parameter to infer potential auditory processing changes in research settings. However, clinical applicability is limited by the variability in P300 latency reference values. Studies describing updated P300 latency reference ranges are lacking.

P300 latency values between 250 and 350ms were established in adults by Kraus et al.^(9)^ however, wider latency ranges, of 220 to 38ms, were also reported.^(5)^ Latency ranges and protocol variability may explain why P300 is not routinely used in clinical practice. Still, P300 is a promising assessment tool that can be employed across several disciplines in different subject areas. Its application as an objective ancillary measure in early recognition of cognitive dysfunction and dementia,^(10)^ assessment of altered emotional states^(11)^ and evaluation of cochlear implants and hearing aids,^(12)^ among other fields, has been described. Therefore, novel studies are warranted to accurately determine normality parameters that may support the routine use of P300 for early recognition of several CNS changes in clinical settings.

## OBJECTIVE

To establish reference intervals for cognitive potential P300 latency using tone burst stimuli.

## METHODS

Twenty-eight subjects aged between 18-59 years were evaluated, 15 of which were men.

The inclusion criteria were as follows: age between 18 and 60 years, normal hearing ability, lack of auditory complaints, no middle ear pathology, no continuous-use medication and ability to understand test procedures. Patients suffering from hearing loss and chronic diseases were excluded.

Patients were submitted to external auditory meatus inspection, pure tone audiometry and acoustic immittance testing prior to cortical potential investigation.

Audiometry was performed in sound-treated booth using Audiotest 330 audiometer. Air conduction thresholds were investigated at 250, 500, 1,000, 2,000, 3,000, 4,000, 6,000 and 8,000Hz using the descending-ascending method. Normal hearing individuals were defined as those with three-frequency (500, 1,000 and 2,000Hz) pure tone averages ≤25 dB HL (decibels hearing level).^(13)^

Tympanometric curve and acoustic reflex assessment was based on acoustic immittance testing performed using middle ear analyzer AT 235 and 226Hz probe tone. Contralateral acoustic reflexes were investigated bilaterally at frequencies of 500 to 4,000Hz. Only subjects with type-A tympanogram and preserved acoustic reflexes were included in the sample.^(14)^

Long latency AEPs were recorded in a silent room using a two-channel device (*Masbe*, Contronic). Patients remained awake throughout the procedure while comfortably seated on an armchair.

The international 10-20 system^(15)^ was used for electrode placement. Surface electrodes were attached to the forehead (Fpz, ground electrode), vertex (Cz, active electrode) and mastoids (reference electrodes M1 and M2, left and right mastoid respectively) with Ten20 conductive paste and micropore tape. Inter-electrode impedance ≤5 Kohms was ensured prior to testing.

Patients were instructed to keep their eyes open, avoid eye movement and note deviant (rare) random stimuli within a series of similar (frequent) stimuli, which should be mentally counted. Stimuli were delivered via ER-3A insert earphones with 80 dB HL intensity.

Frequent and rare stimulus frequency corresponded to 1,000Hz and 2,000Hz tone bursts containing 50 and 100 cycles, respectively, with 20% rise and decay time and trapezoidal envelope. Stimuli were presented in a rare-frequent (oddball) paradigm with 80 and 20% probability respectively. Pulse frequency was 0.8 pulses per second (pps).

P300 waves were investigated using binaural stimulation to prevent patient fatigue. A minimum of two tracings containing 30 rare stimuli each were recorded per patient for increased reliability; tracings were then added together and a single resulting wave acquired. Electrical signals were filtered with 01Hz high-pass and 20Hz low-pass filters. A 1,000ms time window was employed.

P300 latency values were recorded at the highest peak (*i.e*., maximum wave amplitude). The protocol employed for P300 investigation in this study is described in chart 1.

**Chart 1:** P300 investigation protocol

Patient status	Alert
Counting method (rare stimuli)	Mental
Electrode placement	Ground electrode (Fpz), active electrode (Cz), reference electrodes (M1 and M2)
Electrode impedance	≤5 Kohms
Probe	Insert earphones
Stimulation	Binaural
Stimulus intensity	80 dB HL
Presentation paradigm	20% of rare stimuli
	80% of frequent stimuli
Total number of stimuli	300
Frequent stimulus	1,000Hz
Rare stimulus	2,000Hz
Duration	50 cycles (1,000Hz)
	100 cycles (2,000HZ)
Rise and decay time	20%
Envelope	Trapezoidal
Speed	0.8 pps (pulses per second)
Filters	High-pass: 0.1Hz
	Low-pass: 20Hz
Window	1,000ms

Data collected were arranged in spreadsheets using Microsoft Excel software for analysis and comparison. Statistical analyses were performed by an expert statistician. Data normality was assessed using the Lilliefors and the Shapiro-Wilk tests. Variables were compared using the Wilcoxon test. The level of significance was set at 5% (p<0.05), with a 95% confidence interval.

The procedures and assessments in this study were carried out as part of a partnership between a university and the *Clínica Cóser*.

All participants signed the Informed Consent Form (ICF) as proof of their agreement with procedures involved in the study. This project was approved by the Research Ethics Committee (CEP) of *Universidade Federal de Santa Maria* (RS); protocol number CAAE: 25933514.1.0000.5346, committee opinion no. 612.754.

## RESULTS

Mean age of participants was approximately 35 years. Latency and amplitude data of all variables considered are displayed in table 1.

**Table 1:** P300 latency values (ms)

P300	n	Mean	Median	Minimum	Maximum	Standard deviation
RE	28	305.5257	305.7800	248.0800	359.0800	29.45203
LE	28	304.4814	307.1650	243.0300	346.4700	28.06774

RE: right ear; LE: left ear.

P300 component latency did not differ significantly between the left and right ears (Table 2).

**Table 2:** Comparison of P300 latency values recorded from the right and left ears using binaural stimulation

	n	Percent	z score	p value *
P300 RE and P300 LE	23	47.82609	-0.000000	1.000000

*p value (p<0.05).

RE: right ear; LE: left ear; z-score: z-score test.

## DISCUSSION

P300 is currently thought to be a promising assessment tool for detection of central changes. Despite interdisciplinary applicability, P300 continues to have limited use in clinical settings, possibly due to high levels of variability in the test, including latency variability.

Long latency AEPs depend on maturation of peripheral and central structures, a fact that must be taken into account when interpreting test results^(16)^ given the relation between response amplitudes and the number of synapses in the cerebral cortex.^(17)^ Cognitive potential P300 is known to be present in children from 5 to 7 years of age, albeit with reduced amplitude and increased latency, with complete maturation occurring around adolescence.^(5,18)^ Cortical potential values at 14 and 16 years of age are equivalent to those described in adults, according to Steinschneider et al.^(19)^ Participant age in this study (18 years or over) ensured CNS maturation.

P300 latency values of approximately 305ms were documented in this study. Latency values did not differ between the left and right ears; data were therefore grouped and the values compared to similar studies based on standard deviations – *i.e.*, studies reporting similar standard deviations were assumed to be in agreement with findings presented here.

Wave amplitude is thought to be of limited clinical value in P300 assessment; hence, this parameter was not measured. P300 values very widely between studies, both in test and control groups,^(20)^ and may range from 1.7 to 20uV.^(21)^ According to some studies, amplitude is not a relevant parameter in data analysis.^(22,23)^

Mean P300 latency in this trial corresponded to 305ms. Similar P300 latency values (298.1ms) were described in another study^(24)^ involving patients aged 18 to 53 years. Mean P300 values of 313.86, 320 and 298ms (*i.e*., consistent with values in this trial) were also reported elsewhere.^(4,25,26)^ However, discrepant P300 latency values of approximately 341ms (Cz electrode)^(27)^ and 352.2ms^(20)^ have been documented. Participant age in those studies ranged from 7 to 34 years; therefore latency variations may have reflected the maturation process.

Minimum and maximum P300 values in this study (243.03 and 359.08ms) are consistent with previously reported latency ranges of 246 to 361ms,^(24)^ but differ from other ranges (220 to 380ms) described.^(28)^ Still, results of this study are in agreement with P300 ranges documented in the international literature (250 to 350ms),^(9)^ with greater similarities between minimum values. Maximum P300 latency values of 380ms reported by McPherson^(5)^ were not recorded in this trial.

Differences in equipment, patient attention level, patient age, time of data collection and stimulus counting method, among other factors, may explain latency variation between studies.^(28)^ The lack of a consensus and the infrequent use of this test in clinical settings have been pointed out as by Frizzo et al.^(29)^ as potential factors behind the wide variability in cortical potential induction and capture methods among professionals. Electrode placement at Cz or Fz and physiological differences between patients are some of the factors potentially interfering with cortical potential capture. The subjective nature of P300 recognition and sampling may also contribute to the wide variation found in literature.^(6)^

The major scientific contribution of this study lies in the scarcity of updated P300 normative values in literature.^(26,30)^ Data presented are expected to aid clinicians in the interpretation of test results, provided similar parameters are used. Further studies, including meta-analyses, are warranted for more accurate determination of P300 reference values, thereby supporting the evidence-based early diagnosis of central auditory changes.

## CONCLUSION

Mean P300 latency values obtained using the protocol described in this study corresponded to 305ms, with minimum and maximum acceptable latency values of 247.5ms and 362.5ms respectively (mean±2 standard deviation).
